# Antibacterial and Antifungal Activities of the Leaf Exudate of *Aloe megalacantha* Baker

**DOI:** 10.1155/2020/8840857

**Published:** 2020-09-30

**Authors:** Demoze Asmerom, Tesfay Haile Kalay, Gebrehiwot Gebremedhin Tafere

**Affiliations:** ^1^Department of Medicinal Chemistry, School of Pharmacy, College of Health Sciences, Mekelle University, P.O. Box 1871, Mekelle, Ethiopia; ^2^Department of Pharmacognosy, School of Pharmacy, College of Health Sciences, Mekelle University, P.O. Box 1871, Mekelle, Ethiopia; ^3^Department of Pharmacology and Toxicology, School of Pharmacy, College of Health Sciences, Mekelle University, P.O. Box 1871, Mekelle, Ethiopia

## Abstract

Infectious diseases caused by fungi and bacteria are among the major causes of illness and death worldwide. This is mainly implicated by the antimicrobial resistance of the current treatment regimens. Since plant products are house stores of bioactive compounds, it is essential to screen plant-based antimicrobials to come up with novel medicines that counter the grave consequences of antimicrobial resistance. In the folk medicine of Ethiopia, *Aloe megalacantha* is used for the treatment of wound, dandruff, malaria, diabetes, impotence, colon cleansing, amoeba, ascariasis, abdominal pain, urine retention, snake bite, and evil eye. Hence, the present study was aimed to evaluate the antibacterial and antifungal effects of the leaf exudate of *Aloe megalacantha*. Agar well diffusion was employed to determine the antibacterial and antifungal effects. Six bacterial strains, namely, *S. aureus* (standard), *S. aureus* (clinical isolate), *E. coli* ATCC 25922 (standard), *E. coli* (clinical isolate), *K. pneumoniae* (standard), and *P. aeruginosa* ATCC 27853 (standard), and four fungal strains such as *C. albicans*, *C. glabrata*, *C. tropicalis*, and *C. krusei* were studied. The leaf exudate showed the highest activity against *C. krusei* with an average zone diameter of 22.49 ± 0.47 mm at 400 mg/mL. Among the bacterial species, *S. aureus* ATCC 29213 (standard) was the most sensitive with an average zone of diameter of 16.63 ± 0.12 mm at 200 mg/mL. Thus, the present findings support the folklore use of *Aloe megalacantha* for the treatment of different microbial infections.

## 1. Introduction

Infectious diseases caused by fungi and bacteria are among the major causes of illness and death worldwide. Those consequences are mainly implicated by the dramatic rise of antimicrobial resistance of the current treatment regimens [1]. The terrifying aspect of antimicrobial resistance is not limited to a specified geographical area; instead, it affects the entire globe because no one is immune to antimicrobial resistance [2]. Globally, the popular resistant pathogenic organisms which are accompanied with increased morbidity and mortality include *Escherichia coli* (*E. coli*), *Staphylococcus aureus (S. aureus*), methicillin-resistant *S. aureus* (MRSA), vancomycin-resistant *S. aureus*, vancomycin-resistant *enterococci*, *Enterococcus* species, *Klebsiella pneumoniae* (*K. pneumoniae*), *Pseudomonas aeruginosa*, *Streptococcus pneumonia*, *Mycobacterium tuberculosis*, *Salmonella* species, *Acinetobacter*, and *Neisseria gonorrhoeae* [3, 4]. These species pose a fear to the global community because they have been showing a sign of escape from the coverage of the existing antibiotic agents [4].

On the other hand, fungi species that belong to *Candida*, *Cryptococcus*, *Pneumocystis*, and *Aspergillus* are the main causes of illness and death [5]. Of these fungal species, *Candida* species are among the leading causes of superficial and severe life-threatening systemic infections, especially for people living with HIV/AIDS [6]. Most cases of oral candidiasis and esophageal candidiasis are caused by *Candida albicans* (*C. albicans*). However, there is also a dramatic increase in the frequency of fungal infections caused by the emerging non-*albicans Candida* (NAC) species, including *Candida tropicalis* (*C. tropicalis*), *Candida glabrata* (*C. glabrata*), *Candida parapsilosis* (*C. parapsilosis*), and *Candida krusei* (*C. krusei*) [7].

Currently, numerous antibacterial and antifungal agents mainly derived from microbial sources are available in the market [8]. However, the development of antimicrobial resistance becomes a challenge for the existing agents [9]. Due to the reason that plant materials are endowed with essential metabolites which render an important scaffold for the development of potential drug candidates [10], it is relevant to screen plant-based antimicrobials from species that have strong scientific and traditional claims to combat the global concern of antimicrobial resistance.

Since ancient times, the gel and dried leaf exudates of *Aloe* species have been used as mainstay therapy for both humans and animals in various parts of the world [11]. In Ethiopia, where more than 80% of the population depends on traditional medicines for primary health care [12], *Aloe* is among the prominently utilized plant species to manage various conditions [13]. Likewise, *Aloe megalacantha*, which is located in Ethiopia, Eritrea, and Somalia [14], is used for the management of various illnesses. The local communities of northern and eastern Ethiopia use the leaf exudate and the root part of *Aloe megalacantha* for the treatment of malaria, diabetes, impotence, wound healing, dandruff, amoeba, ascariasis, abdominal pain, urine retention, snake bite, evil eye, and colon cleaner [15–17]. Besides, the people of Sidama utilized the leaf of *Aloe megalacantha* for the management of tuberculosis [18]. In vivo and in vitro studies of the leaf exudate of *Aloe megalacantha* also revealed significant antimalarial [19], wound healing, anti-inflammatory [20], antidiabetic, and antihyperlipidemic activities [21, 22]. Numerous compounds isolated from the roots of this plant also displayed cytotoxic effects [23].

Despite those facts, however, the antibacterial and antifungal effects of *Aloe megalacantha* have not been explored yet. Hence, this preliminary study was aimed at investigating the antibacterial and antifungal effects of the leaf exudate of *Aloe megalacantha* against clinically isolated and standard bacterial and fungal strains by using the agar well diffusion technique.

## 2. Materials and Methods

### 2.1. Chemicals and Equipment

The following chemicals were employed to perform the study. The microbial media Sabouraud dextrose agar (SDA), Mueller–Hinton agar (MHA), and dextrose were procured from HiMedia Laboratories, India (Mumbai, India), whereas nutrient agar (NA) was obtained from Thermo Fisher Scientific (Basingstoke, England). Ceftriaxone disk (30 *µ*g) was purchased from Abtek Biologicals (Liverpool, England), and ceftazidime disk (30 *µ*g) was acquired from HiMedia Laboratories (Delhi, India). Ketoconazole (99.5%) and chloramphenicol (99.8%) were obtained from Addis Pharmaceutical Factory (Adigrat, Ethiopia). 0.5 McFarland equivalence/standard was obtained from Ayder Comprehensive Specialized Hospital (Mekelle, Ethiopia). Methylene blue was acquired from Sisco Research Laboratories (Delhi, India). Dimethyl sulfoxide (DMSO) (99.9%) was obtained from Unichem (Maharashtra, India). Petri dish (90 mm and 100 mm) was obtained from Thermo Fisher Scientific, Basingstoke, England). Incubator, UV lamp, autoclave, biosafety cabinet I (BSC I), and vortex mixer were obtained from Camlab, Cambridgeshire, England).

### 2.2. Collection and Identification of Plant Material

The fresh leaf exudate of *Aloe megalacantha* was collected from Wukro, Tigray region, northern Ethiopia (located at 13° 47ʹ north latitude and 39° 35ʹ east longitude) in January 2019. The plant sample was identified and authenticated by a botanist, Dr. Getnet Masresha, and the sample specimen was deposited at the herbarium unit of the Department of Biology, College of Computational and Natural Science, University of Gondar, for future reference with the voucher number of DA0051/2018.

### 2.3. Preparation of the Exudate

The leaf exudate of *Aloe megalacantha* was collected by cutting the leaves transversally near the base and inclining on a stainless tray. The leaf exudate was then exposed in the open shade area for four consecutive days in order to evaporate the water.

### 2.4. Microbial Strains

Six bacterial strains (reference and clinical isolates) and four *Candida* species (clinical isolates only) were obtained from the Department of Microbiology, Mekelle University. The bacterial strains include *S. aureus* ATCC 29213, *S. aureus* (clinical isolate), *E. coli* ATCC 25922, *E. coli* (clinical isolate), *K. pneumoniae* ATCC 700603, and *P. aeruginosa* ATCC 27853. The clinically isolated *Candida* species were *C. albicans*, *C. glabrata*, *C. tropicalis*, and *C. krusei.*

### 2.5. Media Preparation and Inoculum Standardization

The microbial medium was prepared and used according to the manufacturers' directions and specifications. The microbial turbidity of each species was prepared and standardized by the Clinical and Laboratory Standard Institute (CLSI) guideline [24]. The bacterial species were subcultured in nutrient agar (NA), while the *Candida* species were subcultured in Sabouraud dextrose agar (SDA). 0.5 McFarland standard was employed to balance the turbidity of the bacterial and *Candida* inocula.

### 2.6. Antibacterial and Antifungal Assay

#### 2.6.1. Agar Well Diffusion

The antibacterial and antifungal sensitivity tests of the exudate were undertaken by following previous investigations with adaptation [25]. In short, the actively growing bacterial and *Candida* broth cultures were standardized to a density of 0.5 McFarland standard. The balanced inoculum of bacterial species was streaked on the sterile Mueller–Hinton agar (MHA) plate in a 100 mm diameter of sterile petri dish. The uniform thick lawn growth of the seeded media was then allowed to dry at room temperature for about 30 minutes. The media for the *Candida* species were MHA supplemented with 2% glucose and 0.5 *µ*g/mL methylene blue [26].

On each plate, the wells were punched using a 6 mm diameter sterilized borer and assigned with numbers corresponding to the leaf exudates. Then, 50 *μ*L of 200 mg/mL, 100 mg/mL, and 50 mg/mL of the solutions of the leaf exudates dissolved in 1% dimethyl sulfoxide (DMSO) were filled into the corresponding wells. Ceftriaxone (30 *µ*g) disk was used as a positive control for all bacterial strains, except for *P. aeruginosa* ATCC 27853 where ceftazidime (30 *µ*g) was used. The positive control used for the *Candida* was 50 *µ*L ketoconazole dissolved in the DMSO solution. Afterward, the plates were left undisturbed for about 2 hours at room temperature to give sufficient time to diffuse on the inoculated agar, and the plates were transferred into an incubator.

After 24 hours of stay in the incubator, the diameter of the zone of inhibition was measured using a metal caliper and recorded in millimeters (mm). The experiment was carried out in triplicate for each bacterium and *Candida*. The average zone of inhibition was calculated for each test sample and the standard antibiotics.

### 2.7. Preliminary Phytochemical Screening

The qualitative phytochemical screening of the leaf exudate of *Aloe megalacantha* was conducted to assess for the presence of antimicrobial secondary metabolites. It was conducted based on standard techniques described elsewhere [27–29].

### 2.8. Statistical Analysis

SPSS version 21 was employed for statistical analysis. The data were described as mean ± standard error of the mean (SEM). The differences between means of all parameters were performed by using one-way analysis of variance (ANOVA) followed by the Tukey post hoc test. *P* < 0.05 was considered statistically significant.

### 2.9. Results

#### 2.9.1. Antibacterial Activity

As can be seen in Table 1, the antibacterial activities of the leaf exudate of *Aloe megalacantha* were tested using the agar well diffusion technique at a concentration of 50, 100, and 200 mg/mL. The maximum average zone of inhibition was 16.63 mm against standard *S. aureus* ATCC 29213, followed by 15.67 mm for clinically isolated *S. aureus* at a concentration of 200 mg/mL. However, the clinical isolates *E. coli* and standard *K. pneumonia* ATCC 700603 were among the least susceptible species with an average inhibition of 13.03 mm and 13.9 at 200 mg/mL concentration of the leaf exudate, respectively.

### 2.10. Antifungal Activity

**Figure 1:**
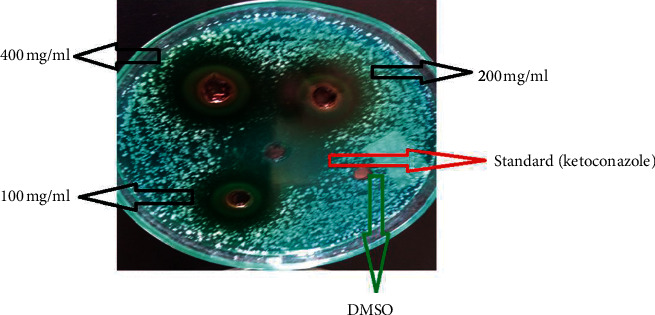
ZI of the leaf exudate of *Aloe megalacantha* against clinically isolated *C. krusei*.

Similar to the antibacterial activity, the antifungal assay was carried out by using the agar well diffusion method, and the growth of all test *Candida* species was inhibited by the tested concentrations of the leaf latex of *Aloe megalacantha* (Table 2). Maximum mean zone of inhibition was observed at 400 mg/mL against *C. krusei* followed by *C. albicans* with a mean diameter of 22.49 mm and 18.48 mm, respectively. The mean zone of inhibition of the leaf exudate of *Aloe megalacantha* against *C. krusei* showed comparable activity with the standard ketoconazole at 400 mg/mL and 200 mg/mL (Figure 1).

### 2.11. Preliminary Phytochemical Screening of Leaf Exudate of *Aloe megalacantha*

The result of the qualitative phytochemical screening of the leaf exudate of *Aloe megalacantha* assured the presence of anthraquinones, polyphenols, cardiac glycosides, flavonoids, alkaloids, saponins, terpenoids, and tannins.

## 3. Discussion

Since the advent of the miracle drug penicillin, in 1928 by Sir Alexander Fleming, antibiotics saved countless lives around the world [30]. However, parallel to the advent of lifesaving antibiotics, the life-threatening antibiotic resistance has emerged [9]. The present study would, therefore, offer a clue for the plant-based antimicrobial discovery to fight the global menace of antimicrobial resistance. Fortunately, the leaf exudate of *Aloe megalacantha* showed relevant activity against both bacterial and *Candida* species. In agreement with the current study, our previous work on the leaf latex and thin-layer chromatography fractions of *Aloe adigratana* Reynolds against those tested microorganisms showed relevant antimicrobial activity [31]. The general antibacterial and antifungal actions could be due to the presence of the bioactive secondary metabolites including anthraquinones, polyphenols, flavonoids, alkaloids, saponins, terpenoids, and tannins. For a long time, it has been known that the mentioned secondary metabolites have possessed relevant antimicrobial properties [10].

The leaf exudate of *Aloe megalacantha* displayed important activity against both Gram-positive and Gram-negative bacterial species in a dose-dependent manner. Although the clinically isolated bacterial strains were resistant to the positive control, ceftriaxone, their growth was inhibited by the leaf exudate of *Aloe megalacantha*. This is not surprising that the clinically isolated strains might have had past exposure to antibiotics, including ceftriaxone. And, microorganisms that had possessed previous exposure to antibiotics are more resistant than those without exposure [32]. Notably, the Gram-negative strain, *E. coli*, is reported to be resistant to the commonly prescribed antibiotics in Ethiopia [33]. Due to this fact, Enterobacteriaceae species including *E. coli* are among the prioritized species which are mandated to the research community to investigate new agents to fight the resistance crisis.

The Gram-positive bacteria, standard *S. aureus* ATCC 29213, was found relatively as the most susceptible pathogen. Similar to this study, the leaf extracts of *Aloe elegans* showed the highest activity against *S. aureus* [34]. This could be due to the absence of additional permeability barriers which efflux the entry of external invaders [35]. *S. aureus* is one of the primary causes of bacteremia and infective endocarditis as well as osteoarticular, skin and soft tissue, pleuropulmonary, and device-related infections [36]. Moreover, the emergence and development of methicillin-resistant *S. aureus* (MRSA) and vancomycin-resistant *S. aureus* (VRSA) make the bacteria more challenging [37]. Similar to this study, most of the leaf extracts of the Ethiopian *Aloe* species like *Aloe trichosantha*, *Aloe sinana*, and *Aloe trigonantha* displayed important antibacterial activity against *S. aureus, S. typhi* Typ 2, *E. coli*, *V. cholerae*, and *Shigella* [38–40]. Besides, Aloe vera, the well documented *Aloe* species, possessed antibacterial activity against *Bacillus subtilis*, *Pseudomonas aeruginosa*, *S. aureus*, *Salmonella typhi*, *K. pneumoniae*, and *E. coli* [41]. Hence, this result is a clue for further investigation of *Aloe megalacantha* and other *Aloe* species.

In the last few decades, there is a growing concern in the number of fatal opportunistic infections as a result of immune-suppressing diseases. Following these diseases, the existing and emerging azole-resistant *Candida* species pose a fear to the global community [42]. To counter this, the antifungal effect of the leaf exudate of *Aloe megalacantha* was assessed, and it displayed a fascinating antifungal effect. Particularly, the leaf latex showed the highest activity against *C. krusei* with an average zone of inhibition of 22. 49 mm (Table 2). This effect is important to remedy the global threat of antifungal resistance. Especially, *C. krusei* is a known pathogen mainly associated with invasive candidiasis, mainly due to its intrinsic resistance to the repeatedly prescribed drug, fluconazole [43]. Also, the effect of the leaf exudate against *C. albicans*, *C. tropicalis*, and *C. glabrata* was highly significant because those species are among the commonly recorded species which pose a global fear. In line with this investigation, Ethiopian *Aloe* species such as *Aloe trigonantha*, *Aloe trichosantha*, and *Aloe elegans* exhibited antifungal activities [38–44]. This might be because *Aloe* species are the house store of various bioactive compounds with diverse biological activities [45].

## 4. Conclusion and Future Prospects

In the present study, the leaf exudate of *Aloe megalacantha* exhibited relevant antibacterial and antifungal activities. From the bacterial species, *S. aureus* ATCC 29213 (standard) was found to be the most sensitive bacterium. However, the clinically isolated bacteria *E. coli* was found to be the least inhibited pathogen. In case of the *Candida* species, the highest activity was observed on *C. krusei*. The general antibacterial and antifungal effects could be due to the presence of anthraquinones, polyphenols, flavonoids, alkaloids, saponins, terpenoids, and tannins which are known to possess significant antimicrobial activity. Thus, the data obtained from the present findings support the folklore use of *Aloe megalacantha* against microbial infections.

Since the current work is a preliminary study, further investigation is required to isolate and characterize the bioactive compounds present in the leaf exudate of *Aloe megalacantha* to offer lead compounds that probably encourage the future arena of plant-based antimicrobial discovery and development.

## Figures and Tables

**Table 1:** Zone of inhibition of the leaf exudate of *Aloe megalacantha* against bacterial species.

Bacterial strains	Diameter of zone of inhibition in mm				
	Latex (mg/mL)			Ceftriaxone 30 *µ*g	DMSO
	200	100	50		
*S. aureus* ATCC 29213 (standard)	16.63 ± 0.12^a2b1c2^	14.23 ± 0.53^a2c2^	11.5 ± 0.16^a2^	22.34 ± 0.734 0	
*S. aureus* (clinical isolate)	15.67 ± 0.26^a3b2c1^	12.46 ± 0.29^a3c3^	10.98 ± 0.3^a2^	14.11 ± 0.61	0
*E. coli* ATCC 25922 (standard)	15.45 ± 0.23^a2b2c2^	11.36 ± 0.71^a2c3^	9.66 ± 0.91^a2^	24 ± 0.28	0
*E. coli* (clinical isolate)	14.48 ± 0.28^a2b1c2^	10.8 ± 0.32^a2c3^	9.53 ± 0.32^a2^	0.00 ± 0.00	0
*P. aeruginosa* ATCC 27853 (standard)	15.66 ± 0.04^a2b2c2^	12.43 ± 0.6^a2c2^	9.57 ± 0.74^a2^	27.03 ± 0.31^*∗*^	0
*K. pneumonia* ATCC 700603 (standard)	15.00 ± 0.12^a2b2c2^	11.6 ± 0.61^a2c3^	10 ± 0.21^a2^	22.8 ± 0.27	0

Values are expressed as mean ± SEM (*n* = 3), and analysis was carried out with one-way ANOVA followed by the Tukey test; ^a^compared to the positive control, ^b^to 100 mg/mL, and ^c^to 50 mg/mL, ^1^*P* < 0.05, ^2^*P* < 0.01, and ^3^*P* < 0.05. 0 = negative control has shown no antibacterial activity; ^*∗*^ceftazidime 30 *µ*g.

**Table 2:** Zone of inhibition of the leaf exudate of *Aloe megalacantha* against *Candida* species.

Fungal strains	Diameter of zone of inhibition in mm				
	Latex (mg/mL)			Ketoconazole 50 *µ*L	DMSO
	400	200	100		
*C. albicans*	18.48 ± 0.58^a2b1c2^	16.55 ± 0.4^a2c3^	15.05 ± 0.34^a2^	24.8 ± 0.17	0
*C. glabrata*	16.23 ± 0.53^a2b1c2^	14.42 ± 0.09^a2c3^	13.21 ± 0.1^a2^	20.71 ± 0.32	0
*C. tropicalis*	17.3 ± 0.58^a2b2c2^	14.99 ± 0.33^a2c3^	13.47 ± 0.77^a2^	25 ± 0.33	0
*C. krusei*	22.49 ± 0.47^a3b2c2^	19.29 ± 0.16^a3c1^	17.04 ± 0.24^a1^	21.1 ± 0.72	0

Values are expressed as mean ± SEM (*n* = 3), and the analysis was carried out with one-way ANOVA followed by the Tukey test; ^a^compared to the positive control, ^b^to 200 mg/mL, and ^c^to 100 mg/mL, ^1^*P* < 0.05, ^2^*P* < 0.01, and ^3^*P* < 0.05. 0 = negative control has shown no antifungal activity.

## Data Availability

The datasets used to support the findings of this study are available from the corresponding author upon request.
